# Relative Survival Following TEER for Significant Mitral Regurgitation: A Contemporary Cohort Analysis

**DOI:** 10.3390/jcm14217825

**Published:** 2025-11-04

**Authors:** Marcel Almendarez, Isaac Pascual, Beatriz Nieves, Rut Alvarez Velasco, Alberto Alperi, Rebeca Lorca, Carmen de la Hoz, Victor Leon, Luis Carlos Zamayoa, Ismael Rivera, Angela Herrero, Pablo Avanzas

**Affiliations:** 1 Heart Area, Hospital Universitario Central de Asturias, Avenida de Roma S/N, 33011 Oviedo, Spainbeanieves21@hotmail.com (B.N.); carmenhoz@hotmail.com (C.d.l.H.); angieherrero@hotmail.com (A.H.);; 2 Health Research Institute of Asturias, Avenida de Roma S/N, 33011 Oviedo, Spain; 3 Faculty of Medicine, University of Oviedo, 33006 Oviedo, Spain; 4 Centro de Investigación en Red de Enfermedades Cardiovasculares (CIBERCV), 28029 Madrid, Spain

**Keywords:** transcatheter edge-to-edge repair, relative survival, mitral regurgitation

## Abstract

**Background/Objectives:** Mitral regurgitation (MR) is the most common valvular defect worldwide, with an increasing incidence attributed to the aging population. Transcatheter edge-to-edge repair (TEER) is a viable treatment, but its long-term survival impact, particularly across sexes, remains underexplored. We aimed to assess relative survival (RS) and excess mortality (EM) in patients undergoing TEER for significant MR, with a focus on sex-based differences. **Methods:** We analyzed 253 patients treated with TEER between October 2015 and August 2024, stratified by sex. Observed survival (OS) was calculated using the actuarial life table method; expected survival (ES) was estimated via the Ederer II method using matched population data. Primary endpoints were RS and EM; secondary endpoints included mortality differences by MR subtype. **Results**: OS at 1, 2, and 3 years was 88.9%, 87.4%, and 78.9%, respectively. EM peaked in the first year (7.8%) and declined thereafter. RS was lower than in the general population, primarily due to persistently reduced RS and elevated EM in men. Women achieved RS comparable to matched peers from year one. Sex was not an independent predictor of mortality (HR 0.88, 95% CI 0.38–2.03, *p* = 0.771). **Conclusions**: In patients with significant MR undergoing TEER, EM was concentrated in the first year. Women reached RS comparable to the general population, while men showed persistent excess mortality. Sex was not independently associated with survival after adjustment.

## 1. Introduction

The most prevalent valvular defect is mitral regurgitation (MR), with a worldwide prevalence of 2%. Recent meta-analyses estimate that up to 0.057% of the general population presents moderate-to-severe MR, a figure that is expected to continue rising due to the aging population [1]. Furthermore, recent evidence suggests that conservative management of moderate-to-severe MR is associated with a 1.67-fold and 2.36-fold increase in mortality, respectively, compared to individuals without MR. This elevated risk was present in all age groups and MR subtypes, underscoring the importance of timely treatment [2].

The recent publication of the 2025 ESC/EACTS guidelines for the management of valvular disease has marked a significant conceptual shift regarding MR classification, with a distinction of organic MR (OMR) (i.e., structural abnormalities of the valve) and functional MR (FMR), with a new subclassification of Atrial MF (AMR), primarily driven by left atrial enlargement and mitral anulus dilation with preserved left ventricle ejection fraction (LVEF) and ventricular MR (VMR), resulting from left ventricle dilation, regional wall abnormalities, reduced LVEF and leaflet tethering [3]. This distinction is crucial for tailoring therapeutic strategies, especially when considering transcatheter edge-to-edge repair (TEER) candidacy. Patients with AMR tend to be older women with atrial fibrillation (AF) and have better long-term prognosis, whereas patients with VMR tend to be male patients with ischemic heart disease and have a worse prognosis [4].

The indications for TEER have broadened in recent years, driven by strong outcomes of randomized trials and large-scale registries. TEER is now considered a viable option for patients with OMR who are at high or prohibitive surgical risk and present with favorable valve anatomy, offering significant improvements in quality of life and a low incidence of adverse events [5]. Both the American (ACC/AHA) and European (ESC/EACTS) guidelines recommend TEER as a first-line therapy for FMR, particularly in cases of VMR without coronary artery disease, who remain symptomatic despite optimal medical treatment [3,6]. This recommendation is based on compelling evidence, specifically the COAPT trial, which was the first to demonstrate a significant reduction in heart failure hospitalizations and deaths due to any cause. The impact of these results was somewhat polarized by the simultaneous publication of the MITRA-FR trial, which showed no significant differences in the same outcomes. After careful evaluation of said trials, the key differences were in patient selection. The COAPT trial enrolled patients with smaller left ventricle size and larger mitral regurgitation compared to the MITRA-FR, with more advanced stages of the disease, with larger ventricle size and less significant MR [7,8]. The latest RESHAPE-HF-2 trial enrolled patients with a similar profile to the COAPT and showed a significant reduction in HF hospitalization and cardiovascular death, showing a reduction in cardiovascular death and heart failure (HF) hospitalizations when specific anatomical criteria are met [9,10].

Despite the 2025 ESC guidelines introducing new considerations, they still lack specific sex-based treatment recommendations [3]. Furthermore, there are significant gaps in the evidence regarding pathophysiology, indications, and timing of treatment. Women often present at advanced stages of mitral valve disease and remain consistently underrepresented in both surgical and TEER trials [11]. Women undergoing MitraClip therapy tend to be older, more frequently present with AMR, and display distinct anatomical and procedural profiles compared to men. These sex-specific differences have a crucial impact on clinical outcomes [4].

These differences are not merely demographic, while life expectancy is generally recovered in patients with OMR undergoing surgical valve repair, data suggest that women may experience less complete recovery in survival outcomes compared to men. These differences may be attributed to later referrals, distinct anatomical features, and persistent underrepresentation in clinical trials. Regarding TEER, data from the MitraSwiss registry, which included patients treated from 2011 to 2018 and primarily using the third-generation MitraClip device, show that women aged 60 to 89 years achieved a 5-year relative survival rate of 97.4%, closely matching that of age-matched controls [12]. Similar survival restoration was also observed in male patients aged 80 to 89 years. However, patients with FMR had a residual excess mortality that persisted regardless of sex, highlighting the relation of ventricular pathology and therapeutic response [13].

The impact of TEER on life expectancy recovery in patients with significant mitral regurgitation remains insufficiently explored, particularly in contemporary cohorts treated with the latest-generation MitraClip device. Taking advantage of the fact that our hospital serves as the reference for this therapy throughout the province, we can compare the relative survival rates of patients of the same age, sex, and geographic area and determine whether patients treated with TEER achieve a life expectancy comparable to that of the general population on long-term follow-up.

## 2. Materials and Methods

### 2.1. Sample and Study Design

This is a retrospective analysis of our institutional database of all consecutive cases that underwent TEER for symptomatic significant MR, from October 2015 to August 2024. We included patients with OMR, AMR, and VMR who received optimal medical treatment and were deemed better candidates for this procedure after careful examination by a heart team comprising at least a clinical cardiologist, a cardiac surgeon, an interventional cardiologist specializing in TEER, and a specialist in cardiovascular imaging. Our center serves as the reference hospital for a population of 1 million inhabitants, with a team of over ten years of experience. Exclusion criteria were being < 18 years old and not being able to sign an informed consent.

All patients were treated using the MitraClip^®^ device (Abbott Vascular, Santa Rosa, CA, USA; EU). With initial patients using older generations, however, most cases were performed using the fourth-generation device, which featured an array of four sizes and independent grippers to enable more precise leaflet grasping. The procedural steps have been described previously [5]. Procedural success was defined as placement of one or more clips resulting in a post-interventional MR severity of ≤2+.

We constructed the reference population using mortality tables provided by the National Institute of Statistics of Spain (INE). This institution publishes mortality data on its official website, stratified by age range, sex, and autonomous region in Spain. To compare our sample of patients with the reference population, we matched our data with the mortality tables provided by the INE, using subjects of the same age, sex, and geographical area [14]. All the subjects were followed for at least one year after the main event, finalizing the recruitment in August 2024. We treated our patients during admission and selected the optimal medical treatment at discharge, following the latest available European Society of Cardiology guidelines at the moment of discharge and follow-up.

### 2.2. Data Recollection and Follow-Up

Data on baseline characteristics, procedure, hospitalization, and discharge were obtained from a dedicated database prospectively collected from all patients undergoing TEER. Patients had predetermined follow-up visits with clinical revision at 3 and 6 months, and a combined clinical and echocardiographic evaluation 12 months after the procedure. Any additional data was readily available from electronic clinical history and phone interviews for patients without sufficient information.

Our database was divided into two groups based on sex, and we constructed a reference population using mortality tables from INE. Standardized endpoints and causes of death were defined according to the Valve Academic Research Consortium 3 consensus document, as well as the Mitral Valve Academic Research Consortium criteria [15,16].

### 2.3. Primary Endpoints

To determine the RS and EM of symptomatic patients with significant MR despite OMT undergoing TEER.

### 2.4. Secondary Endpoints

- To determine if there were sex related differences regarding RS and EM of symptomatic patients with significant MR despite OMT undergoing TEER.
- To determine if sex was an independent predictor of mortality on the long-term follow-up.

### 2.5. Statistical Analysis

Quantitative variables were expressed as mean ± standard deviation or median and interquartile range (IQR) and compared using Student’s *t*-test and the Wilcoxon rank-sum test, respectively. Categorical variables were expressed as numbers (percentages) and compared using χ^2^ and, in the case of several categories, using the ANOVA test.

We performed a multivariable Cox regression analysis to identify factors predicting mortality in the general population, stratified by sex and different MR classifications (OMR, AMR, and VMR). The variables introduced were defined using a backward stepwise method with a cutoff *p*-value < 0.10. Associations were expressed as hazard ratios (HR) with 95% confidence intervals (95% CI). The model’s predictive capacity was evaluated using Harrell’s C test and survival analysis with Kaplan–Meier curves. Differences were statistically significant, with a *p*-value of <0.05.

To estimate relative survival and excess mortality of patients undergoing TEER and compare them to the general population, we used the Ederer II method. The following concepts are detailed to clarify our results [17,18].

1. Observed survival (OS): This refers to the cumulative probability of survival from all causes in the studied cohort of patients with significant MR undergoing TEER. It is estimated using the actuarial life table method and expressed with a 95% confidence interval (CI), providing a statistical range of the survival probability over time.
2. Expected survival (ES): This represents the survival probability of the reference population matched by age, sex, and geographic region, but free from MR. It reflects the baseline mortality risk in the absence of the underlying valvular pathology. Our cohort of patients is included within the reference population but represents a negligible proportion. ES was calculated using the Ederer II method, which maintains individuals at risk until death or censoring. This approach is the widely preferred method, particularly in studies with follow-up durations of ten years or less, due to its conservative estimation and reduced risk of survival overestimation. If the ES curve lies within the 95% CI of OS, no statistically significant difference in survival is assumed, suggesting potential restoration of life expectancy following TEER. Moreover, in retrospective analyses such as ours, accurately determining the cause of death can be inherently biased and unreliable. In this context, RS estimates offer a distinct advantage, as they enable researchers to assess mortality attributable solely to the disease under study without requiring precise information on the specific causes of death [17,18].
3. RS: The ratio of OS in the MR cohort to the ES of the matched general population. It isolates disease-specific mortality by estimating survival under the hypothetical condition that patients could only die from MR or its consequences. An RS of 100% indicates no EM attributable to MR, whereas an RS of 90% implies a 10% reduction in survival due to the disease. If the 95% CI of RS includes 100%, the difference is not statistically significant, suggesting that TEER may have effectively restored the survival impact of MR.
4. EM: It is calculated as 1—RS. An EM of 10% denotes that 10% of patients died due to MR or its consequences, whereas an EM of 0% implies restoration of survival. If the 95% CI of EM includes 0, the EM is considered not statistically significant.

All analyses were performed using STATA /IC 15.1 (STATA Corp, College Station, TX, USA). The “strs” command (Version 1.4.3.1), as described by Dickman et al., was used to estimate the OS, ES, and RS according to the Ederer II method [17,18,19]. To assess the competing risk analysis, we used the stcrreg command and the stcurve to visualize the competing risk regression [20].

## 3. Results

### 3.1. Baseline Characteristics

From October 2015 to August 2024, 253 patients presenting with symptoms despite OMS and significant MR were referred by the Heart Team to TEER (Supplementary Material Figure S1). The median age was 75.4 years, and 163 cases (64.4%) were male. Men were younger (73.7 vs. 77.6; *p* = 0.026), and presented with more comorbidities like diabetes (40.5% vs. 25.8%; *p* = 0.02), smoking habit (10% vs. 44.8%; *p* ≤ 0.001), chronic kidney disease (50.9% vs. 35.6%; *p* = 0.018), coronary artery disease (54% vs. 36.7%; *p* = 0.008), previous coronary artery bypass grafting (16.6% vs. 4.4%; *p* = 0.005), peripheral vascular disease (13.4% vs. 4.4%; *p* = 0.002) and previous implantable cardioverter defibrillator (23.9% vs. 11.1%; *p* = 0.013).

Regarding the mechanism of MR, 203 cases (80.2%) corresponded to VMR, 30 patients (11.9%) to AMR, and 20 (7.9%) to OMR, without significant differences among the sexes. Baseline left ventricle ejection fraction was significantly lower for men with a mildly reduced LVEF of 38.9 ± 12.2, whereas most women presented a preserved LVEF of 57.1 ± 11.9 (*p* < 0.001). The remaining baseline characteristics are presented in Table 1.

### 3.2. Procedure and Hospitalization

Over 84.4% of patients were treated with the 4th-generation MitraClip, with a significant difference between men and women (76.5% vs. 98.9%; *p* < 0.001). Initial cases were mainly male with a prohibitive surgical risk and were treated with the first three generations. On the other hand, women had a lower surgical risk and were included in later stages, as the indication for MitraClip had already expanded. Procedural success was achieved in 214 (84.6%) cases without differences related to sex. Procedural complications were similar; however, during hospitalization, men presented a lower incidence of acute coronary syndrome (7.8% vs. 0%; *p* < 0.001) and acute kidney injury (2.5% vs. 10%; *p* = 0.009). The detachment rate was relatively high at 21 (8.3% of cases); however, this accounted for all detachments, including those that were resolved with a second or third clip during the procedure. If only detachments on the follow-up were accounted for, a total of 9 devices were detached (3.5%). Hospitalization times were short, with a median duration of 1 day, as most procedures were elective, and patients were typically discharged the day after surgery. There was a total of 8 deaths during hospitalization, similar for both sexes. The remaining procedural and hospitalization data are presented in Table 2.

### 3.3. Long-Term Outcomes

The mean follow-up was 3.3 ± 2.4 years. During this period, a total of 54 patients (21.3%) died, and only 199 patients reached the end of the follow-up period. The crude mortality rate for men was higher than that for women: 43 (26.4%) vs. 11 (12.2%), *p* = 0.009. Over 50% of the deaths occurred during the first year, 43 (26.4%) for men and 11(12.2%) for women (*p* = 0.075). A total of 11 cardiovascular deaths (37.9%) occurred in the first year, and 12 (48%) occurred after the first year. The rest were non-cardiovascular causes. The most frequent cause of death was heart failure with nine cases (31%), followed by cancer in eight cases (27.6%). Female sex, as assessed through competing risk analysis, showed a significantly lower cardiovascular mortality compared to males when considering non-cardiovascular mortality as a competing event (HBR: 0.19, IC95% 0.05–0.9; *p* = 0.03). However, when additional covariates were included in the model (HBR 0.37, IC95% 0.07–2.1; *p* = 0.262), this difference was no longer statistically significant (Supplementary Material Figure S2). Details of long-term outcomes are shown in Table 3. Details of the causes of death can be found in Supplementary Materials Table S1.

**Figure 1:**
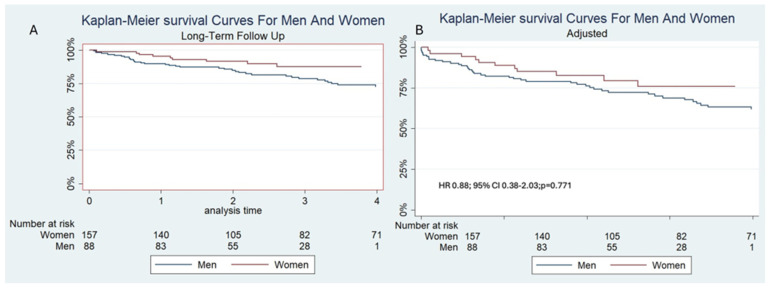
(**A**): Unadjusted and (**B**): Adjusted Kaplan–Meier survival curves for men and women, showing that sex was not a predictor of mortality.

In the univariable analysis, sex approached statistical significance as a predictor of mortality, with a hazard ratio (HR) of 0.51 and a 95% confidence interval (CI) of 0.26–1.00 (*p* = 0.053) (Figure 1). However, when adjusting for them in the multivariable analysis, sex was not an independent risk factor, HR 0.88, 95%CI 0.38–2.03 (*p* = 0.771) (Figure 1). There were no predictors of mortality in the multivariable analysis. Univariable and multivariable analyses are presented in Table 4.

### 3.4. Observed and Expected Survival

**Figure 2:**
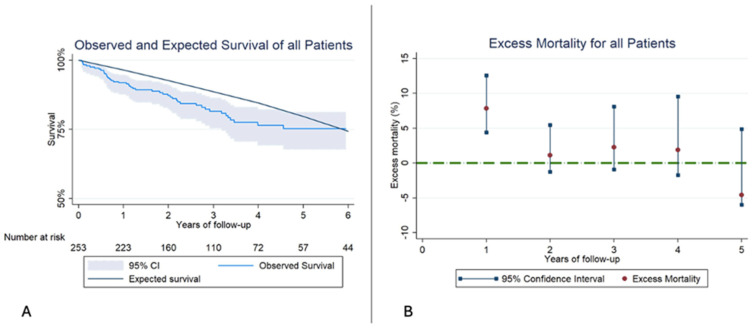
(**A**): Kaplan–Meier observed survival (OS) and expected survival (ES) of all patients. The reference population’s ES is greater than our sample’s OS. (**B**): Excess mortality (EM) for all patients. In the first year of follow-up the EM is higher than the reference population, but in the following years, EM stays similar.

The OS for all patients at 1,2, and 3 years of follow-up was 88.9% (95% CI 84.3–92.2%), 87.4% (95% CI 79.1–88.38%), 78.9% (95% CI 72.8–83.8%), and the ES for the same period was 96.4%, 92.6% and 88.6%. The ES was not included in the 95% CI of the OS, indicating a reduced RS among all patients from the same sex, age, and region compared to the general population. The interval specific EM mortality was significantly higher during the first year of 7.8% (95% CI 2.6–12.8%); however, after the first year, EM remained similar to the general population,1.1% (95% CI −1.3–5.5%) and 2.3% (95% CI −0.9–8.1%) at 2 and 3 years (Figure 2, Table 5). 

**Figure 3:**
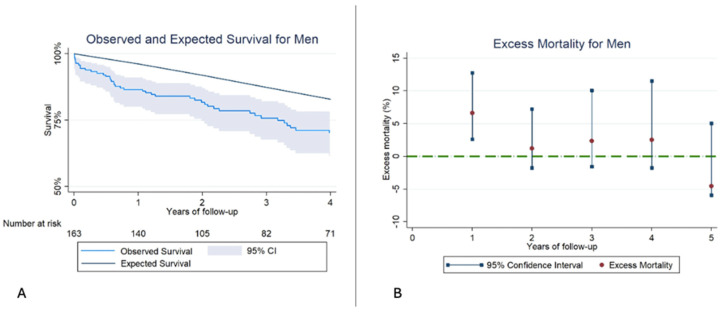
(**A**): Kaplan–Meier observed survival (OS) and expected survival (ES) for Men. The ES of the reference population remains above the OS of our sample. (**B**): Excess mortality (EM) for men. The EM remains high during the first year, reaching similar levels in the subsequent years of follow-up.

When analyzing men, we obtained similar results. There was reduced RS compared to healthy men from the general population of their same age and geographic area. EM was significantly higher during the first year; however, after the first year, EM levels remained similar to those of men from the general population (Figure 3, Table 6).

**Figure 4:**
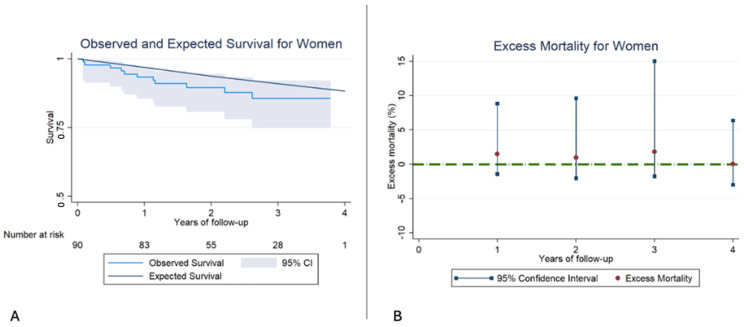
(**A**): Kaplan–Meier observed survival (OS) and expected survival (ES) for women. In this case, the ES falls within the 95% confidence interval, and therefore, no differences exist between the OS of our sample and the ES of the reference population. (**B**): Excess mortality (EM) for women. In this case, there are no significant differences in EM in any of the years of follow-up.

Interestingly, women undergoing TEER presented a similar RS compared to the general population. Furthermore, EM remained low and not statistically significant from the beginning of the follow-up (Figure 4, Table 7).

## 4. Discussion

The main findings of this analysis were that symptomatic patients with MR, despite OMT undergoing TEER, presented a reduced RS compared to the general population of the same age, sex, and geographical area in a follow-up of 3.4 ± 2.4 years. Furthermore, there was an interval-specific EM of 7.8% during the first year. After this period, EM remained unchanged, similar to the reference population. For men, the same pattern was followed, with a reduced RS and an EM of 6.6% during the first year. On the contrary, female patients presented a similar RS, and there was no EM from the first year of follow-up (Central Figure). In other words, TEER was effective in recovering life expectancy compared to women of the general population of the same age and region. Finally, male sex tended to predict mortality in the univariable analysis, but this association was not statistically significant in the multivariable analysis.

In this analysis, the main indication for TEER was FMR in over 80% of patients, mainly in those who were younger and had a higher burden of comorbidities. In contrast, women were older and had higher prevalences of OMR and AMR. These results are consistent with those reported in leading real-world registries, such as the GIOTTO trial and German TRAMI. They differ from the MitraSwiss registry and the American TVT registry, where more than half of the patients were OMR [21,22,23,24].

Contemporary randomized trials, such as CLASP IID, have demonstrated that one-year outcomes following TEER are favorable and comparable between men and women in terms of survival and freedom from major adverse events. Nonetheless, essential nuances in baseline characteristics were observed: female patients presented with fewer comorbidities but were more symptomatic at baseline. Additionally, a significant interaction between sex and the rate of freedom from adverse events at one year was noted in the MitraClip arm. However, after adjusting for baseline differences, female patients achieved safety and effectiveness outcomes comparable to those of male patients (86.0% vs. 85.9%, *p* = 0.985). These findings align with our own results, which also show a tendency toward sex-related differences in both the univariable and competing risk analyses. However, after adjusting for baseline characteristics and other covariates in the multivariable model, sex was no longer identified as an independent predictor of outcomes [25].

Despite differences between men and women, procedural success was achieved in 84.6% of cases, and a similar number of clips was implanted, with a low overall burden of complications during hospitalization, an incidence of stroke < 1%, bleeding < 3% and significant vascular complications <3%. Detachment occurred in 8.3%, which is higher than reported in most trials (0.8% in the GIOTTO trial and 1.3% in the TVT trial) [21,23]. However, this data considers partially detached devices detected during the procedure, and in most cases, procedural success was achieved with a second or third device.

Women had a higher prevalence of acute coronary syndrome and acute kidney injury before discharge, but this did not have an impact on hospitalization times (median of 1 day) or in-hospital mortality (3.2%). Hospitalization times were significantly higher in most trials, with a median of 9 days in the German TRAMI trial, 5 days in the GIOTTO trial, and 5 days for the MitraSwiss registry, all with similar in-hospital mortality rates of around 3%. It is worth noting that this is a contemporary cohort, with over 80% of patients treated with the 4th-generation MitraClip device, contrary to the largest registries, which report data from mostly the third generation or older [21,22,23,24].

After one year, most patients presented in NYHA class I or II, with no meaningful differences between sexes, indicating comparable baseline functional status. Additionally, sustained results were observed after TEER, with only 5.9% of patients exhibiting mitral regurgitation greater than grade 2. All-cause mortality was 11.5% at one year and 21.3% at the end of follow-up, with a numerically higher rate for men (26.4%) compared to women (12.2%). Cardiovascular deaths accounted for almost half of the events. Mortality in our trial was lower at one year and three years compared to the trials mentioned above [21,22,23,24]. After a multivariable Cox regression analysis, Sex was not found to be an independent predictor of mortality.

Using data from the MitraSwiss registry, Biasco L et al. analyzed 1140 patients from 2011 to 2018, evaluating them using relative survival analysis according to age and MR etiology. The results showed that 50.8% of patients had OMR and 45% had FMR. They presented a cumulative RS of 91.1% (95% CI: 82.5–98.6%) for OMR with an EM of 8.9% and of 71.5% (95% CI: 63.0–79.3%) in FMR with a cumulative EM of 28.5% at 5 years, respectively. When considering patients aged 80–89 years, the cumulative RS was 93% (95% CI: 83.3–101.9%), without significant differences compared to the general Swiss population [13]. These results are similar to those obtained in the surgical treatment of PMR, where life expectancy may be recovered in selected patients [26]. Most patients in our cohort presented FMR, and our survival rates were very similar, at 72.7% at the end of follow-up.

In a second analysis by the same author stratified by sex and MR etiology, RS for all patients, men, and women was 80.5% (95% CI, 74.6–86.0), 84.9% (95% CI, 75.8–92.9), and 78.0% (95% CI, 70.2–85.3), respectively [12]. We observed similar results for men, showing persistently reduced ES compared to age-matched peers, with OS declining from 89.8% at 1 year to 78.7% at 3 years, and a significant EM in year one (6.6%; 95% CI, 2.6–12.8%), which penalized the cumulative RS. In contrast, women recovered relative survival early, maintaining OS of 88.3%, 91.4%, and 87.1% across 1, 2, and 3 years, with consistently low and non-significant EM during follow-up. However, these results should be interpreted with caution, as the study was designed to detect changes in the entire cohort compared to the general population, and the sample size may be insufficient to detect sex-related differences.

Several factors may influence these findings, including the use of the fourth-generation MitraClip, which introduced several pivotal enhancements that directly impact procedural safety and efficacy. These include the expansion to four clip sizes, among them wider clips designed to improve leaflet grasping in cases with significant coaptation gaps and complex lesions. The addition of independent gripper actuation offers operators greater control and precision during deployment, while the redesigned delivery catheter facilitates smoother navigation and clip placement. Collectively, these innovations broaden the therapeutic applicability of TEER, particularly in anatomically challenging scenarios [5]. Furthermore, we included a more contemporary cohort, as well as the predominance of FMR, all of which may influence procedural success and long-term outcomes.

These findings suggest that sex-specific factors should be considered when evaluating candidacy and timing for TEER, particularly in patients who may benefit from earlier intervention or closer post-procedural monitoring. The future of mitral TEER is promising, with increasingly favorable outcomes being achieved through meticulous patient selection in cases involving complex mitral anatomies (e.g., significant coaptation gaps, commissural lesions, multiple regurgitant jets, or prior failed TEER). Transcatheter mitral valve replacement (TMVR) may offer a viable alternative and could play a complementary role alongside TEER in patients deemed unsuitable for surgical repair [27].

### Limitations

This study is a retrospective observational analysis conducted at a single center in Spain, which may limit the generalizability of the results. The sample size is relatively modest, and although the cohort reflects real-world practice with contemporary TEER techniques, caution is warranted when extrapolating these results, as the statistical power was not specifically designed for these outcomes. Nevertheless, they may influence patient selection and results. Similarly, we could not explore OS, EM, and RS according to MR etiology. Consequently, larger, multicenter studies are needed to examine sex-specific outcomes and long-term survival following TEER. Our results should be interpreted as hypothesis-generating for future trials to seek external validation.

## 5. Conclusions

In this contemporary cohort of patients with significant mitral regurgitation undergoing TEER, overall survival remained lower than that of the general population, with EM concentrated in the first year. Notably, women achieved RS rates comparable to those of their matched peers, suggesting that TEER may restore life expectancy in select patients. Although men exhibited persistent EM, especially within the first year, these findings highlight the importance of sex-aware evaluation and tailored follow-up strategies. Overall, the results reinforce TEER as a safe and effective therapy with the potential to improve long-term outcomes in high-risk MR populations.

## Figures and Tables

**Table 1:** Baseline characteristics.

Variable	Total (n = 253)	Female (n = 90)	Male (n = 163)	*p*
Age, years (IQR)	75.4 (70.1–81.3)	77.6 (71–82.6)	73.7 (69.5–80.5)	0.026
BMI, kg/m^2^ ± SD	27.8 ± 8.1	27.6 ± 6.4	27.9 ± 8.9	0.724
Hypertension	186 (73.5%)	63 (70%)	123 (75.5%)	0.346
Diabetes	89 (35.3%)	23 (25.8%)	66 (40.5%)	0.020
Dyslipidemia	129 (51%)	43 (47.8%)	86 (52.8%)	0.447
Smoking Habit				
Current Smokers	82 (32.4%)	9 (10%)	73 (44.8%)	<0.001
Ex-smokers	37 (14.6%)	9 (10%)	28 (17.2%)	<0.001
CKD	115 (45.5%)	32 (35.6%)	86 (50.9%)	0.018
CAD	121 (43.1%)	33 (36.7%)	88 (54%)	0.008
Previous PCI	77 (30.4%)	24 (26.7%)	53 (32.5%)	0.333
Previous CABG	31 (12.3%)	4 (4.4%)	27 (16.6%)	0.005
Stroke/TIA	30 (11.9%)	7 (7.8%)	23 (14.1%)	0.136
PVD	34 (13.4%)	4 (4.4%)	30 (13.4%)	0.002
Atrial fibrillation	164 (64.8%)	56 (62.2%)	108 (66.3%)	0.519
Pacemaker	22 (8.7%)	8 (8.9%)	14 (8.6%)	0.935
ICD	49 (19.4%)	10 (11.1%)	39 (23.9%)	0.013
ICD/CRT	33 (13%)	7 (7.8%)	26 (16%)	0.064
COPD	55 (21.8%)	16 (18%)	39 (23.9%)	0.274
Previous Annuloplasty	5 (2%)	2 (2.2%)	3(1.8%)	0.834
Baseline NYHA class				
NYHA I	14 (5.5%)	2 (2.2%)	12 (7.4%)	0.087
NYHA II	67 (26.5%)	32 (35.6%)	35 (21.4%)	0.015
NYHA III	113 (44.6%)	38 (42.2%)	75 (46%)	0.562
NYHA IV	59 (23.3%)	18 (20%)	41 (25.2%)	0.353
Baseline Echocardiography				
Baseline LVEF, %	45.4 ± 14.9	57.1 ± 11.9	38.9 ± 12.2	<0.001
Baseline MR grade				0.067
Grade 3	44 (17.5%)	21 (23.3%)	23 (14.2%)	
Grade 4	208 (82.5%)	69 (76.7%)	139 (85.8%)	
Atrial MR	30 (11.9%)	14 (15.6%)	16 (9.8%)	0.176
Ventricular MR	203 (80.2%)	67 (74.4%)	136 (83.4%)	0.176
Organic MR	20 (7.9%)	8 (8.9%)	12 (7.4%)	0.667

Variables are represented as mean ± standard deviation for quantitative variables, number (%) for categorical variables, and median (interquartile range) for variables with uneven distribution. Abbreviations: BMI (Body Mass Index), CABG (Coronary Artery Bypass Grafting), CAD (Coronary Artery Disease), CKD (Chronic Kidney Disease), COPD (Chronic Obstructive Pulmonary Disease), CRT (Cardiac Resynchronize Therapy), ICD (Implantable Cardioverter Defibrillator), LVEF (Left Ventricle Ejection Fraction), MR (Mitral Regurgitation), NYHA (New York Heart Association), PCI (Percutaneous Coronary Intervention), PVD (Peripheral Vascular Disease), TIA (Transitory Ischemic Accident).

**Table 2:** Periprocedural outcomes.

Variable	Total (N = 253)	Female (N = 90)	Male (N = 163)	*p*
		Procedural		
Urgent/emergent procedure	77 (30.4%)	26 (28.9%)	51 (31.3%)	0.691
Number of clips				0.166
- 1	124 (49%)	40 (44.4%)	84 (51.3%)	
- 2	115 (45.5%)	42 (46.7%)	73 (44.8%)	
- 3	14 (5.5%)	8 (8.9%)	6 (3.7%)	
4th generation device	211 (84.4%)	87 (98.9%)	124 (76.5%)	<0.001
Final MR grade <3	229 (90.5%)	84 (93.3%)	145 (89%)	0.2554
Final MR gradient < 5 mmHg	234 (7.5%)	13 (7.6%)	19 (7.5%)	0.705
Procedural Success	214 (84.6%)	75 (83.3%)	139 (85.3%)	0.682
		Procedural Complications		
Detachment	21 (8.3%)	9 (10%)	12 (7.4%)	0.467
Vascular complication				
- Major	6 (2.37%)	4 (4.4%)	2 (1.2%)	0.107
- Minor	1 (0.4%)	1 (1.1%)	0	0.176
Embolization	2 (0.8%)	1 (0.6%)	1 (0.8%)	0.669
Tamponade	2 (0.8%)	0	2 (1.2%)	0.291
		Hospitalization		
Stroke/TIA	1 (0.4%)	1 (1.1%)	0	0.176
AKI	13 (5.1%)	9 (10%)	4 (2.5%)	0.009
ACS	7 (2.8%)	7 (7.8%)	0	<0.001
Bleeding	7 (2.8%)	4 (4.4%)	3 (1.8%)	0.227
Death	8 (3.2%)	2 (2.2%)	6 (3.7%)	0.526
Hospitalization days, median (IQR)	1 (1–3)	1 (1–2)	1 (1–3)	0.174

Variables are represented as mean ± standard deviation for quantitative variables, number (%) for categorical variables and, median (interquartile range) for variables with uneven distribution. Abbreviations: ACS (Acute Coronary Syndrome), AKI (Acute Kidney Injury), MR (Mitral Regurgitation), TIA (Transient Ischemic Attack).

**Table 3:** Long-term outcomes.

Variable	Total (N = 253)	Female (N = 90)	Male (N = 163)	*p*
Mean follow-up, years ± SD	3.3 ± 2.4	2.4 ± 1.2	3.7 ± 2.7	<0.001
Deaths in the first 30 days of follow-up	7 (2.77%)	0 (0%)	7 (2.77%)	0.046
Deaths in the 1st year of follow-up	29 (11.5%)	6 (6.7%)	23 (14.1%)	0.075
Deaths in the long-term follow-up	54 (21.3%)	11 (12.2%)	43 (26.4%)	0.009
CV deaths	23 (9.1%)	2 (2.2%)	21 (12.9%)	0.005
Non-CV Deaths	33 (13%)	9 (10%)	24 (14.7%)	0.286
HF hospitalizations at the end of the follow-up	61 (24.1)	21 (23.3%)	40 (20.5%)	0.830
NYHA class at 12 months				
NYHA I	138 (61.6%)	47 (56%)	91 (61.6%)	0.581
NYHA II	62 (27.7%)	25 (29.8%)	37 (26.4%)	0.369
NYHA III	22 (9.8%)	11 (13.1%)	11 (7.9%)	0.139
NYHA IV	2 (0.9%)	1 (1.2%)	1 (0.7%)	0.669
MR grade > 2	15 (5.9%)	6 (6.7%)	9 (5.5%)	0.712
MR gradient > 5	62 (24.5%)	21 (23.3%)	41 (25.2%)	0.747
LVEF, % ±SD	44.9 ±13.6	46.3 ± 13.4	44.1 ± 13.8	0.226

Variables are represented as mean ± standard deviation for quantitative variables and number (%) for categorical variables. Abbreviations: CV (Cardio-Vascular), HF (Heart Failure), LVEF (Left Ventricle Ejection Fraction), MR (Mitral Regurgitation), NYHA (New York Heart Association).

**Table 4:** Univariate and multivariable analysis.

Variable	Univariable				Multivariable	
	HR	95% CI	*p*	HR	95% CI	*p*
Sex	0.5	0.3–1	0.053	0.9	0.38–2	0.739
Smoking habit	2	1.2–3.5	0.012	1.6	0.9–2.9	0.117
ICD/CRT	1.6	1–2.4	0.033	1.4	0.9–2.2	0.09
LVEF	0.98	0.9–1	0.027	0.99	0.9–1.1	0.379
Baseline MR Grade 4	2.8	1–7.7	0.048	2.4	0.9–6.8	0.09
HF hospitalizations	0.9	0.5–1.7				
Age	1	1–1.01	0.698			
BMI	1	0.9–1.1	0.458			
Hypertension	1.1	0.8–1.6	0.525			
Dyslipidemia	1.1	0.6–.9	0.775			
Diabetes	1.1	0.6–1.8	0.839			
CKD	1.2	0.7–2	0.512			
Stroke/TIA	0.8	0.3–1.9	0.593			
COPD	1.6	0.9–2.8	0.133			
CCS	0.9	0.5–1.6	0.808			
Previous PCI	1.2	0.7–2.1	0.524			
Previous CABG	1.3	0.7–2.8	0.414			
PVD	1	0.5–2.1	0.991			
Pacemaker	1.3	0.5–3	0.567			
ICD	1.1	0.6–2.2	0.703			
Baseline						
- NYHA	ref					
- NYHA II	0.3	0.9–1.1	0.06			
- NYHA III	0.6	0.2–1.6	0.288			
- NYHA IV	0.6	0.2–1.75	0.333			
Atrial MR	0.7	0.2–1.9	0.450			
Ventricular MR	1.1	0.5–2.2	0.885			
Organic MR	1.4	0.6–3.5	0.480			
Urgent/emergent procedure	1.3	0.7–2.2	0.434			
4th generation MitraClip	0.8	0.4–1.5	0.454			

Abbreviations: BMI (Body Mass Index), CABG (Coronary Artery Bypass Grafting), CCS (Coronary Chronic Syndrome), CKD (Chronic Kidney Disease), COPD (Chronic Obstructive Pulmonary Disease), CRT (Cardiac Resynchronize Therapy), HF (Heart Failure), ICD (Implantable Cardioverter Defibrillator), LVEF (Left Ventricle Ejection Fraction), MR (Mitral Regurgitation), NYHA (New York Heart Association), PCI (Percutaneous Coronary Intervention), PVD (Peripheral Vascular Disease), TIA (Transitory Ischemic Accident).

**Table 5:** Observed survival, expected survival, and excess mortality of all patients.

Year of Follow-Up	Survival of Patients Undergoing TEER (Observed Survival)	Survival in the Reference Group (Expected Survival)	Interval-Specific Excess of Mortality
First year	88.9% (95% CI 84.3–92.2%)	96.4%	7.8% (95% CI 4.4–12.6%)
Second year	87.4% (95% CI 79.1–88.38%)	92.6%	1.1% (95% CI −1.3–5.5%)
Third year	78.9% (95% CI 72.8–83.8%)	88.6%	2.3% (95% CI −0.9–8.1%)
Fourth year	73.9% (95% CI 66.8–79.7%)	84.6%	1.9% (95% CI −1.7–15.1%)
Fifth year	72.7% (95% CI 65.3–78.8%)	79.6%	−4.6% (95% CI −6–4.8%)

Abbreviations: TEER (transcatheter edge-to-edge repair).

**Table 6:** Observed survival, expected survival, and excess mortality for men.

Year of Follow-Up	Survival of Patients Undergoing TEER (Observed Survival)	Survival in the Reference Group (Expected Survival)	Interval-Specific Excess of Mortality
First year	89.8% (95% CI 83.9–93.6%)	96.1%	6.6% (95% CI 2.6–12.8%)
Second year	84.8% (95% CI 78–89.6%)	91.9%	1.2% (95% CI −1.8–7.2%)
Third year	78.7% (95% CI 70.8–84.7%)	87.3%	2.3% (95% CI −1.6–10.1%)
Fourth year	72.7% (95% CI 64–79.7%)	82.8%	2.5% (95% CI −1.8–11.5%)
Fifth year	71.6% (95% CI 62.7–78.7%)	78%	−6% (95% CI −6–5%)

Abbreviations: TEER (transcatheter edge-to-edge repair).

**Table 7:** Observed survival, expected survival, and excess mortality for women.

Year of Follow-Up	Survival of Patients Undergoing TEER (Observed Survival)	Survival in the Reference Group (Expected Survival)	Interval-Specific Excess of Mortality
First year	88.3% (95% CI 88.3–98.3%)	96.9%	1.5% (95% CI −1.4–8.9%)
Second year	91.4% (95% CI 82.7–95.8%)	93.6%	1% (95% CI −2–9.6%)
Third year	87.1% (95% CI 76–93.3%)	90.9%	1.8% (95% CI −1.8–15%)
Fourth year	87% (95% CI 75.9–93.2%)	88.3%	0.1% (95% CI −3–6.4%)

Abbreviations: TEER (transcatheter edge-to-edge repair).

## Data Availability

Data will be made available upon request.

## Supplementary Materials

The following supporting information can be downloaded at: https://www.mdpi.com/article/10.3390/jcm14217825/s1, Table S1: causes of Death; Figure S1: Flowchart of patient selection. Abbreviations: MR: Mitral regurgitation TEER: Transcatheter edge-to-edge repair; Figure S2: Competing risk analysis comparing cardiovascular death versus non-cardiovascular death among men and women. Abbreviations: CV: Cardiovascular.

## Abbreviations

AMR	Atrial mitral regurgitation
EM	excess mortality
FMR	functional mitral regurgitation
INE	National institute of statistics
LVEF	Left Ventricle Ejection Fraction
OMR	organic mitral regurgitation
OMT	optimal medical treatment
OS	observed survival
RS	relative survival
TEER	Transcatheter edge-to-edge repair
VMR	ventricular mitral regurgitation
