# Supplementary material for: Relative Survival Following TEER for Significant Mitral Regurgitation: A Contemporary Cohort Analysis
Source: J Clin Med. 2025 Nov 4;14(21):7825. doi: 10.3390/jcm14217825 (PMC12608293; doi:10.3390/jcm14217825)
Supplement: Supplementary file 1 [file jcm-14-07825-s001.zip › jcm-3901289-supplementary.pdf]

SUPPLEMENTARY MATERIAL

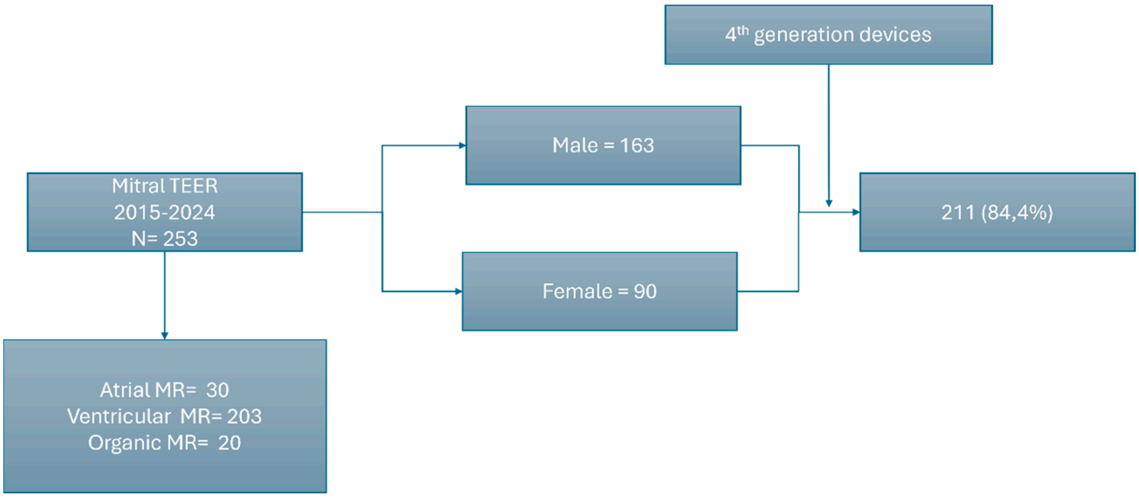

Supplementary material Figure S1: Flowchart of patient selection. Abbreviations: MR: Mitral regurgitation TEER: Transcatheter edge-to-edge repair

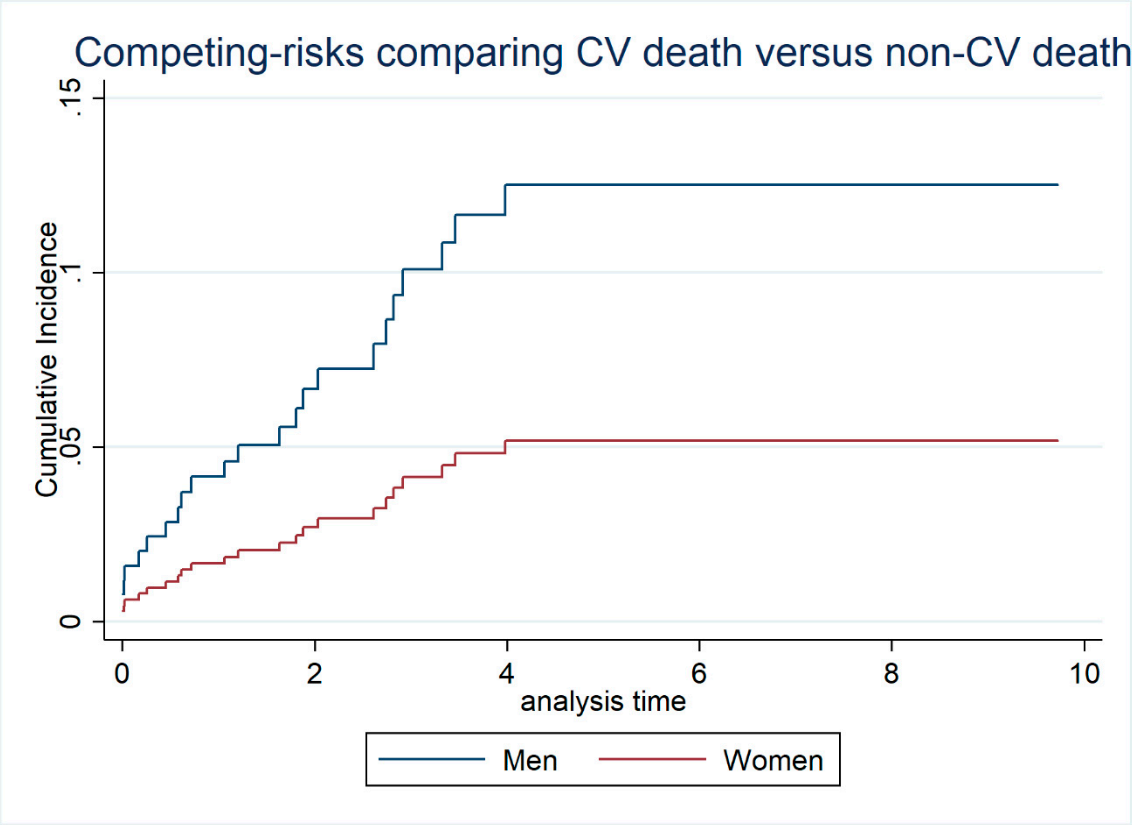

Supplementary material Figure S2: Competing risk analysis comparing cardiovascular death versus non-cardiovascular death among men and women. Abbreviations: CV: Cardiovascular

Supplementary Material Table S1. Causes of Death

Variable	Total (N = 253)	Female (N=90)	Male (N=163)
	**Cardiovascular Death**		
**Acute MI**	1 (1.85%)	0 (0%)	1 (1.85%)
**Sudden Death**	4 (7.41%)	0 (0%)	4 (7.41%)
**Heart Failure**	17 (31.48%)	2 (3.70%)	15 (27.78%)
**Stroke**	0 (0%)	0 (0%)	0 (0%)
**Other CV causes**	0 (0%)	0 (0%)	0 (0%)
	**Non-cardiovascular death**		
**Neoplasia**	2 (3.70%)	0 (0%)	2 (3.70%)
**Pulmonary**	1 (1.85%)	0 (0%)	1 (1.85%)
**Infections**	14 (25.93%)	6 (11.11%)	8 (14.81%)
**Gastrointestinal**	2 (3.70%)	0 (0%)	2 (3.70%)
**Accident/Trauma**	1 (1.85%)	0 (0%)	1 (1.85%)
**Other Organ Failures**	3 (5.56%)	1 (1.85%)	2 (3.70%)
**Other non-CV**	0 (0%)	0 (0%)	0 (0%)
**Gastrointestinal**	2 (3.70%)	0 (0%)	2 (3.70%)
**Accident/Trauma**	1 (1.85%)	0 (0%)	1 (1.85%)
**Unknown**	9 (16.67%)	2 (3.70%)	7 (12.96%)

Causes of death according to the academic research consortium stratified by sex. Abbreviation: CV: Cardiovascular, MI: Myocardial Infarction.
